# Radiolucent Area in the Retromandibular Trigone Area in a Young Asymptomatic Male

**DOI:** 10.1111/odi.70235

**Published:** 2026-07-09

**Authors:** Marco Uccelli, Lucia Ferrari, Luciana Ambrosiani, Paolo Vescovi, Tiziano Testori

**Affiliations:** ^1^ Department of Biomedical Surgical and Dental Sciences University of Milan Milan Italy; ^2^ IRCCS Galeazzi‐Sant'ambrogio Hospital, Milan. Department of Implantology and Oral Rehabilitation University Dental Clinic; ^3^ Department of Pathology Valduce Hospital Como Italy; ^4^ Director of the Department of Pathology Valduce Hospital Como Italy; ^5^ Dir Unt Oral Surgery, Oral Medicine and Laser Therapy University Center of Dentistry‐ Department of Medicine and Surgery University of Parma Italy; ^6^ Head of the Department of Implantology and Oral Rehabilitation. IRCCS Galeazzi‐Sant'ambrogio Hospital, Milan University Dental Clinic; ^7^ Department of Biomedical, Surgical and Dental Sciences University of Milan Milan Italy; ^8^ Department of Periodontics and Oral Medicine University of Michigan School of Dentistry Ann Arbor MI USA; ^9^ Department of Oral Medicine, Infection and Immunity Harvard School of Dental Medicine Boston MA USA

**Keywords:** exploratory flap surgery, histopathological analysis, inflamed mucosa, pathological probing depths, radiolucent area, wound healing

## 1 Case Report

A 35‐year‐old male patient presents with inflamed mucosa and pathological probing depths distal to mandibular molars 3.7 and 4.7, ten years post‐extraction of teeth 3.8 and 4.8. The patient maintains a high level of oral hygiene, which appears inconsistent with the clinically observed inflammatory condition. The patient reports only a mild cough, without other systemic symptoms.

A cone beam computed tomography (CBCT) scan reveals a radiolucent area at the healing sites of previous extractions.

Exploratory flap surgery exposes dense, vascularized tissue with an inflammatory appearance. Tissue samples for histopathological analysis are collected from several regions: an internal portion of the flap, cortical bone, and multiple samples from the medullary component.

After excision, the lingual cortical plate appears intact (Figure 1A,B).

**FIGURE 1:**
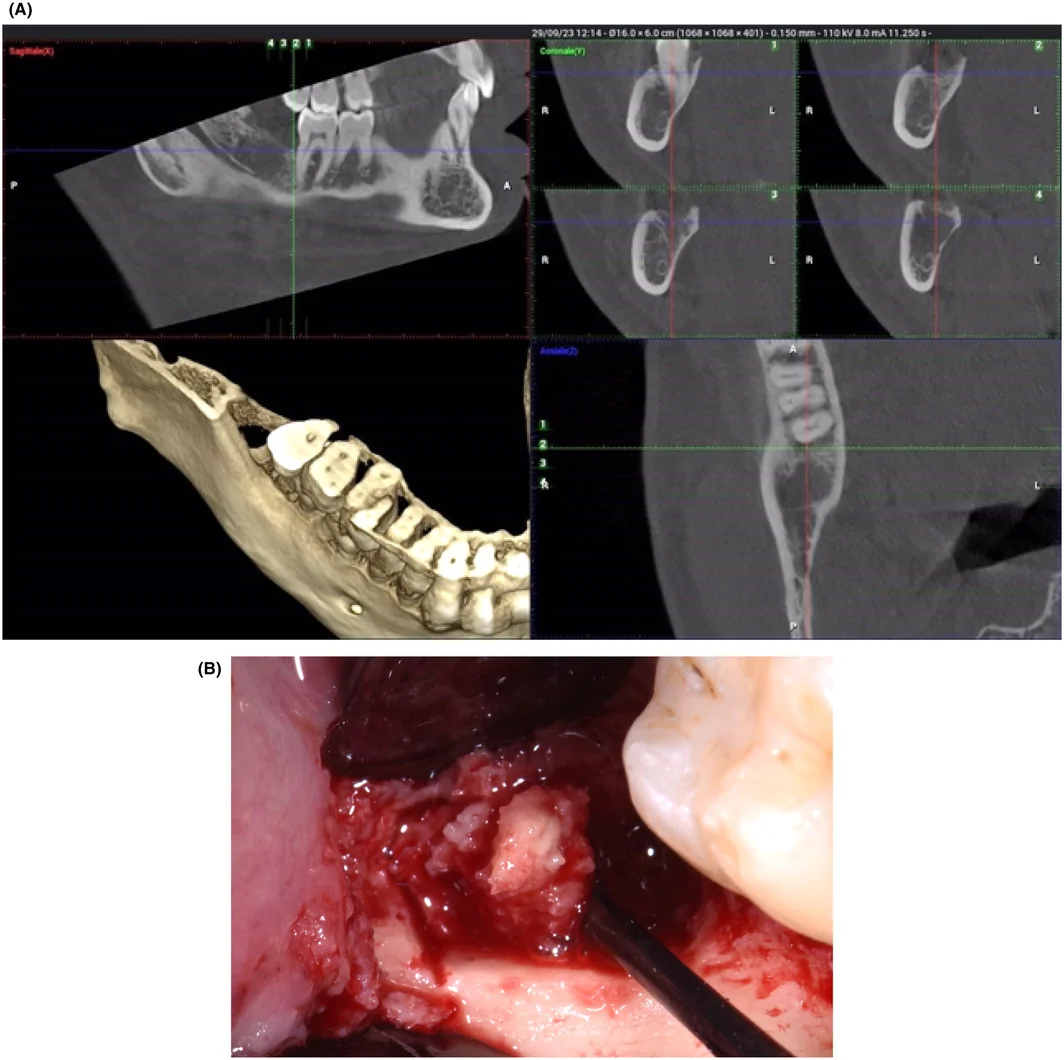
(A) CBCT of the post‐extraction site. (B) Tissue sample for histopathological analysis collected from the medullary component.

**FIGURE 2:**
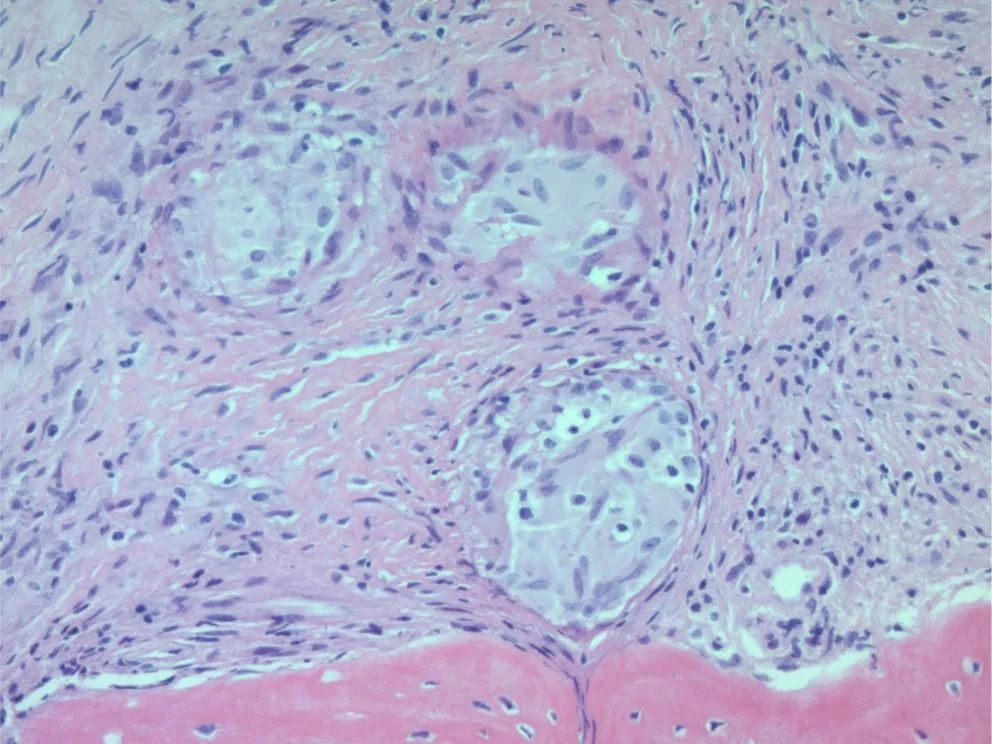
The image shows bone tissue surrounded and infiltrated by diffuse fibrosis containing inflammatory cells and epithelioid granulomas.

## 2 What Is Your Diagnosis?

Based on the patient's history, physical examination, and computed tomography images, which one of the following is the most probable diagnosis?

- Tuberculosis
- Crohn's disease
- Orofacial granulomatosis
- Oral sarcoidosis

## 3 Diagnosis

Answer D: Oral sarcoidosis. Multiple biopsies were performed, and histopathological examination demonstrated many non‐caseating granulomas within fibrous connective tissues.

The diagnostic complexity of oral granulomatous lesions lies in distinguishing between sarcoidosis, Crohn's disease, and orofacial granulomatosis (OFG), as all three conditions can present with non‐caseating granulomas within oral tissues (Alamoudi et al. 2024; Roychowdhury et al. 2024; Alawi 2005). However, various clinical, radiological, and histopathological factors strongly support the diagnosis of sarcoidosis in this case.

The radiolucency observed on computed tomography, extending from the maxilla, aligns with documented cases of intraosseous sarcoidosis, which may present as an ill‐defined lesion without cortical expansion (Reddy et al. 2016). Histological analysis revealed non‐caseating granulomas composed of epithelioid macrophages within a fibrous connective tissue matrix rich in lymphocytes, a hallmark feature of sarcoidosis (Alamoudi et al. 2024; Roychowdhury et al. 2024; Alawi 2005). Negative staining for PAS and Grocott effectively rules out infectious granulomatous diseases such as tuberculosis, which typically exhibit caseous necrosis (Alawi 2005; Reddy et al. 2016).

To differentiate sarcoidosis from Crohn's disease and OFG, both systemic and localized manifestations must be considered. Crohn's disease primarily affects the gastrointestinal tract, and while it may present with oral symptoms, these generally follow gastrointestinal manifestations such as abdominal pain, diarrhea, or weight loss (Judson 2021). The absence of gastrointestinal involvement in this patient renders Crohn's disease an unlikely diagnosis (Reddy et al. 2016; Judson 2021).

Orofacial granulomatosis typically presents with swelling of the lips, buccal mucosa, and facial soft tissues, but rarely involves the maxilla (Alamoudi et al. 2024; Roychowdhury et al. 2024). Furthermore, OFG is generally confined to the orofacial region without systemic manifestations, whereas sarcoidosis often presents as a multisystemic disease with potential pulmonary, lymphatic, ocular, and cutaneous involvement (Alamoudi et al. 2024; Judson 2021, 2015).

Histologically, sarcoid granulomas tend to be larger and more well‐defined than those observed in Crohn's disease or OFG (Alawi et al. 2020). This characteristic was evident in the present case, where the granulomas appeared well‐formed and distinct. Further systemic evaluation, including serum angiotensin‐converting enzyme (S‐ACE) levels, chest radiography, and pulmonary function tests, can provide additional confirmation of sarcoidosis (Judson 2021, 2015; Alawi et al. 2020).

Considering the histopathological findings, the absence of infectious organisms, and the lack of gastrointestinal or localized facial involvement, the diagnosis of oral sarcoidosis emerges as the most consistent with the clinical presentation (Alamoudi et al. 2024; Roychowdhury et al. 2024; Alawi 2005; Judson 2021). This case highlights the importance of including sarcoidosis in the differential diagnosis of oral granulomatous lesions, particularly when maxillary involvement suggests a possible systemic disease. Early recognition is essential, as sarcoidosis may progress to significant systemic complications if left untreated (Alamoudi et al. 2024; Judson 2021, 2015).

## 4 Outcome

After the diagnosis, additional radiographic examinations revealed non‐caseating granulomatous lung lesions, leading to pharmacological therapy, while biopsies of the left inguinal lymph node and nasopharynx showed numerous well‐formed non‐necrotizing granulomas with associated hyaline fibrosis, and a second opinion from the Mayo Clinic (US) concluded that, in the appropriate clinical context, the histopathological findings could be compatible with sarcoidosis Figure 2.

This case exemplifies the importance of further investigation when faced with unexplained clinical and radiographic findings, ensuring accurate diagnosis and significantly improving both the quality and expectancy of the patient's life.

## Author Contributions

**Marco Uccelli:** writing – original draft, writing – review and editing, data curation, investigation. **Lucia Ferrari:** conceptualization, investigation, writing – original draft, validation, methodology, data curation. **Luciana Ambrosiani:** visualization, formal analysis, supervision, writing – review and editing, investigation, validation. **Paolo Vescovi:** formal analysis, supervision, writing – review and editing, validation. **Tiziano Testori:** conceptualization, methodology, investigation, writing – review and editing, formal analysis, supervision, data curation, visualization, validation, project administration.

## Consent

The patient reported in this manuscript provided written informed consent for the publication of the case details.

## Conflicts of Interest

All authors have no conflicts of interest to disclose.

## Data Availability

The data that support the findings of this study are available on request from the corresponding author. The data are not publicly available due to privacy or ethical restrictions.

## References

[odi70235-bib-0001] Alamoudi, W. A. , R. A. Abdelsayed , T. P. Sollecito , G. A. Alhassan , R. Kulkarni , and M. A. Bindakhil . 2024. “Causes of Oral Granulomatous Disorders: An Update and Narrative Review of the Literature.” Head and Neck Pathology 18, no. 1: 72. 10.1007/s12105-024-01678-7. PMC11306859 39110261

[odi70235-bib-0002] Alawi, F. 2005. “Granulomatous Diseases of the Oral Tissues: Differential Diagnosis and Update.” Dental Clinics of North America 49, no. 1: 203–221. 10.1016/j.cden.2004.07.012. 15567369

[odi70235-bib-0003] Alawi, F. , B. E. Shields , T. Omolehinwa , and M. Rosenbach . 2020. “Oral Granulomatous Disease.” Dermatologic Clinics 38, no. 4: 429–439. 10.1016/j.det.2020.05.004. 32892852

[odi70235-bib-0004] Judson, M. A. 2015. “The Clinical Features of Sarcoidosis: A Comprehensive Review.” Clinical Reviews in Allergy & Immunology 49, no. 1: 63–78. 10.1007/s12016-014-8450-y. 25274450

[odi70235-bib-0005] Judson, M. A. 2021. “Granulomatous Sarcoidosis Mimics.” Frontiers in Medicine 8: 680989. 10.3389/fmed.2021.680989. PMC8295651 34307411

[odi70235-bib-0006] Reddy, B. V. , K. K. Kuruba , S. Yalamanchili , and M. Mupparapu . 2016. “Granulomatous Diseases Affecting Jaws.” Dental Clinics of North America 60, no. 1: 195–234. 10.1016/j.cden.2015.08.007. 26614955

[odi70235-bib-0007] Roychowdhury, D. , R. P. Chatterjee , S. Gayen , et al. 2024. “Oral Granulomatous Disorders: A Diagnostic Insight.” Cureus 16, no. 7: e65742. 10.7759/cureus.65742. PMC11360674 39211635

